# HLA class I genes modulate disease risk and age at onset together with *DR-DQ* in Chinese patients with insulin-requiring type 1 diabetes

**DOI:** 10.1007/s00125-021-05476-6

**Published:** 2021-05-22

**Authors:** Ziyu Jiang, Wenqian Ren, Hua Liang, Jinhua Yan, Daizhi Yang, Sihui Luo, Xueying Zheng, Guo-Wang Lin, Yingxin Xian, Wen Xu, Bin Yao, Janelle A. Noble, Jin-Xin Bei, Leif Groop, Jianping Weng

**Affiliations:** 1 Department of Endocrinology and Metabolism, Guangdong Provincial Key Laboratory of Diabetology, The Third Affiliated Hospital of Sun Yat-sen University, Guangzhou, China; 2 Department of Endocrinology of the First Affiliated Hospital, Division of Life Sciences and Medicine, University of Science and Technology of China, Hefei, China; 3 State Key Laboratory of Oncology in South China, Sun Yat-sen University Cancer Center, Guangzhou, China; 4 Children’s Hospital Oakland Research Institute, Oakland, CA USA; 5 Department of Clinical Sciences, Lund University Diabetes Centre, Lund University, Skåne University Hospital, Malmö, Sweden

**Keywords:** Basic science, Genetics of type 1 diabetes, Human

## Abstract

**Aims/hypothesis:**

The study aimed to investigate the effects of HLA class I genes on susceptibility to type 1 diabetes with different onset ages, in addition to the well-established effects of HLA class II genes.

**Methods:**

A total of 361 patients with type 1 diabetes (192 patients with onset <18 years and 169 patients with onset ≥18 years) and 500 healthy control participants from China were enrolled and genotyped for the *HLA-A*, *-B*, *-C*, *-DQA1*, *-DQB1* and *-DRB1* genes using next-generation sequencing.

**Results:**

The susceptible *DR3* (β = −0.09, *p* = 0.0009) and *DR4-DQ8* (β = −0.13, *p* = 0.0059) haplotypes were negatively associated with onset age, while the protective *DR11* (β = 0.21, *p* = 0.0314) and *DR12* (β = 0.27, *p* < 0.0001) haplotypes were positively associated with onset age. After adjustment for linkage disequilibrium with *DR-DQ* haplotypes, A*11:01:01 was positively associated with onset age (β = 0.06, *p* = 0.0370), while the susceptible C*15:02:01 was negatively associated with onset age (β = −0.21, *p* = 0.0050). The unit for β was double square-root (fourth root) transformed years of change in onset age associated with per copy of the HLA haplotype/allele. In addition, B*46:01:01 was protective (OR 0.41, 0.46; *p*c [corrected for multiple comparisons] = 0.0044, 0.0040), whereas A*24:02:01 (OR 2.71, 2.25; *p*c = 0.0003, 0.0002) and B*54:01:01 (OR 3.96, 3.79; *p*c = 0.0018, 0.0004) were predisposing in both the <18 group and the ≥18 group compared with healthy control participants. In the context of *DR4-DQ4*, A*11:01:01 (61.29% vs 28.26%, *p*c = 0.0144) was increased while the predisposing A*24:02:01 (19.35% vs 47.83%, *p*c = 0.0403) was decreased in patients with onset ≥18 years when compared with patients with onset <18 years.

**Conclusions/interpretation:**

In addition to *DR-DQ* haplotypes, novel HLA class I alleles were detected to play a role in susceptibility to type 1 diabetes with different onset ages, which could improve the understanding of disease heterogeneity and has implications for the design of future studies.

**Graphical abstract:**

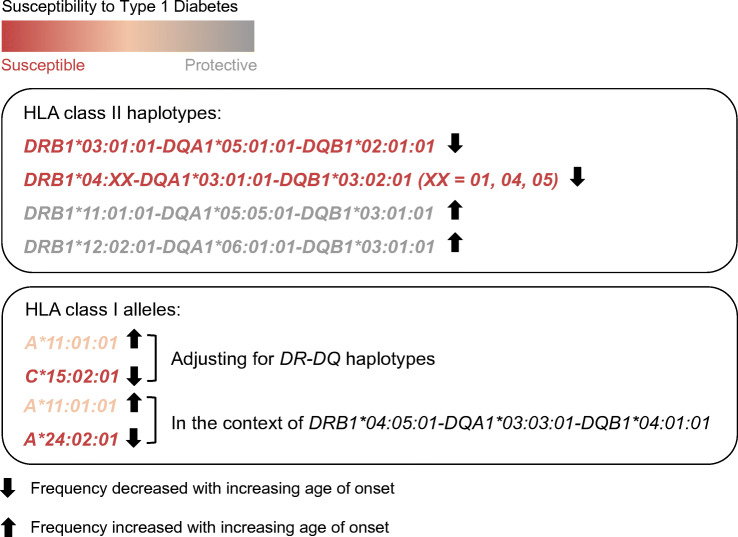

**Supplementary Information:**

The online version contains peer-reviewed but unedited supplementary material available at 10.1007/s00125-021-05476-6.

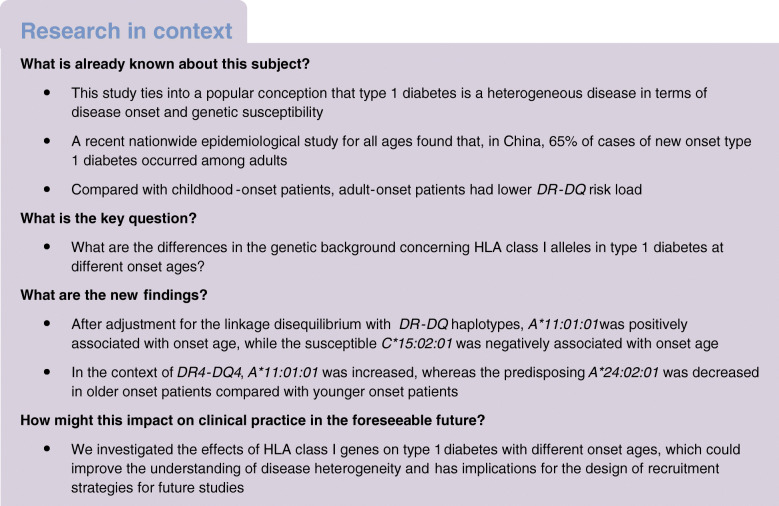



## Introduction

Type 1 diabetes is a chronic autoimmune disease characterised by insulin deficiency due to pancreatic beta cell loss, which leads to hyperglycaemia and lifelong dependence on exogenous insulin [1]. Although the precise aetiology of type 1 diabetes is not completely understood, it is widely acknowledged that type 1 diabetes is a highly heritable disease. The major genetic risk determinants are HLA class II genes (especially *DRB1-DQA1-DQB1* haplotypes), and to a lesser degree, HLA class I genes (*HLA-A*, *-B* and *-C*) [2–5].

Recently, the heterogeneity of type 1 diabetes has been more appreciated. Studies have shown that patients with type 1 diabetes differed in their genetic background, immunopathological process and clinical characteristics [6, 7]. Although type 1 diabetes most often occurs in children and adolescents, it can indeed occur at any age. In China, our recent nationwide epidemiological study for all ages found that almost 65% of the newly diagnosed type 1 diabetes cases occurred in adults [8]. Previous studies showed that compared with childhood-onset patients, adult-onset patients had less aggressive disease onset, less severe clinical manifestation, lower frequency of diabetic autoantibodies and lower *DR-DQ* risk load [9–14]. It has been reported that European patients with younger onset age had a higher frequency of the susceptible DRB1*03:01-DQB1*02:01/DRB1*04 -DQ8 genotype, but a lower frequency of the protective DRB1*15:01-DQB1*06:02 and DRB1*07:01-DQB1*03:03 haplotypes than those with older onset age [14]. Our previous study in Chinese patients showed that, compared with childhood-onset patients, adult-onset patients had a lower frequency of the high-risk *DR3/DR4* and *DR3/DR9* genotypes, but higher frequency of *DR3/DR3* genotype and *DR3/X*, *DR4/X*, or *DR9/X* (X, non-risk) genotypes [11].

In addition to *DR-DQ*, HLA class I genes have been shown to have independent effects on type 1 diabetes [3, 5]. In European populations, after accounting for linkage disequilibrium (LD) with *DR-DQ* haplotypes, A*24:02, B*39:06 and B*18:01 were predisposing to type 1 diabetes, while A*11:01, B*57:01 and B*44:03 were protective [3, 5]. Additionally, previous studies in Europeans showed that A*24:02 and B*39:06 were associated with younger onset age of type 1 diabetes [3, 14]. In Asian populations, A*24:02, B*54:01, B*58:01 and C*03:02 were found associated with increased susceptibility to the disease, whereas B*13:02 and B*15:01 were protective [15, 16]. However, the relationship between HLA class I genes and age-related heterogeneity in type 1 diabetes remains unclear, especially in older onset patients.

Therefore, in this study, by conditioning on HLA class II genes, we aimed to further investigate the genetic differences of HLA class I genes in type 1 diabetes at different onset ages.

## Methods

### Participants

This study enrolled 361 patients with type 1 diabetes from the Guangdong Type 1 Diabetes Translational Medicine Study (GTT Study). We based our inclusion criteria on (1) the ADA descriptions of type 1 diabetes, (2) the WHO reports for the classification of diabetes and (3) the inclusion criteria of the DCCT.

All patients with type 1 diabetes included in our study had to meet the following three criteria:

1. All participants had a clinical diagnosis of type 1 diabetes and were characterised by immediate initiation of insulin treatment at disease onset;
2. They had to meet at least one of the following criteria: (a) symptoms of hyperglycaemia at diagnosis; (b) history of diabetic ketoacidosis (DKA): defined as DKA occurred between disease onset and first visit; (c) tested positive for type 1 diabetes-associated autoantibodies; (d) fasting and stimulated C-peptide levels <200 pmol/l;
3. To avoid misdiagnosis of other types of diabetes like non-insulin-dependent diabetes, and to distinguish from latent autoimmune diabetes in adults (LADA), the diagnostic time window for all participants had to be no less than 18 months.

Details of the follow-up procedures were previously described [8]. During the follow-up visits, patients that were less than 1 year old at the onset of diabetes or not insulin-dependent were further excluded. Additional exclusions included fulminant type 1 diabetes (FT1D) and LADA. Any cases of uncertain diagnoses were submitted to the expert committee for final judgement. We ascertained all cases with a final diagnosis of type 1 diabetes. Based on our previous study, which mainly focused on the effects of *DR-DQ* on the classical type 1 diabetes with different onset ages [11], this study intended to extend the analysis of HLA genes beyond the classical class II genes to investigate the effects of HLA class I genes in a larger dataset and to explore the association of HLA genes with islet autoantibodies in a dataset with autoantibody negative participants included. Therefore, in addition to the cases of classical type 1 diabetes included in our previous study [11], we expanded the sample size without setting additional selection criteria to restrict DKA or autoantibody status. As a result, a total of 361 patients currently with data available were included in this study (electronic supplementary material [ESM] Fig. 1 and ESM Fig. 2).

Control participants consisted of 500 volunteers who were recruited from the Health Check Center at the Third Affiliated Hospital of Sun Yat-sen University and met the following criteria: (1) a normal response to 75 g OGTT; (2) no family history of diabetes or autoimmune diseases. Control participants were matched to cases in terms of region of recruitment and self-reported ancestry (Han Chinese). The control group comprised 229 men and 271 women. The χ^2^ test showed that there was no difference in sex ratio among patients with onset <18 years, patients with onset ≥18 years and control participants (*p* = 0.2164). The median age of the 500 control participants was 39.0 (IQR 31.0–48.0; range 20.0–75.0) years. Power calculation can be found in ESM Table 1.

All participants or their parents signed the informed consent for the project. The study was approved by the Institutional Review Board of the Third Affiliated Hospital of Sun Yat-sen University.

### Definitions and measurements

Clinical metrics including onset age, duration of classic symptoms (polyuria, polydipsia and weight loss) and occurrence of DKA at disease onset were captured from medical records when patients were first diagnosed in local hospitals. Data about demographics and anthropometric measurements, and blood samples (for diabetic autoantibodies, HbA_1c_ and C-peptide measurements) were collected by trained physicians and nurses at the first visit.

Autoantibodies and C-peptide concentrations were centrally tested using standard methods as described previously [17]. Briefly, GAD autoantibodies (GADA), insulinoma-associated antigen-2 autoantibodies (IA-2A) and zinc transporter 8 autoantibodies (ZnT8A) were analysed using fasting serum with radiobinding assay confirmed by the Islet Autoantibody Standardization Program at the First Affiliated Hospital of Nanjing University. Assay sensitivity and specificity for GADA were 64% and 98%, respectively, 64% and 100% for IA-2A, respectively, and 36% and 98% for ZnT8A, respectively. Fasting and postprandial C-peptide levels were measured using an iodine (125I) radioimmunoassay kit (Beijing North Institute of Biological Technology, Beijing, China; reference range: fasting, 200.0–1133.3 pmol/l, and five times after stimulation; intra- and inter-batch coefficients of variation 0.46% and 0.99%, respectively). Postprandial C-peptide was measured at 2 h during the mixed-meal tolerance test. The median diabetes duration at the time of the autoantibody and C-peptide measurements of 361 patients was 1.80 (IQR 0.03–5.29) years.

Based on disease onset age, 361 patients were categorised into two groups: 192 patients with onset <18 years of age (<18 group) and 169 patients with onset ≥18 years of age (≥18 group).

### Genotyping methods

Genomic DNA of 361 patients with type 1 diabetes and 500 control participants was extracted from peripheral blood using DNeasy Blood & Tissue Kit and QIAamp DNA Blood Mini Kit (QIAGEN, Venlo, the Netherlands). Amplicons of HLA class I (*HLA-A*, *-B* and *-C*) and class II (*HLA-DRB1*, *-DQA1* and *-DQB1*) genes were generated in samples from 361 patients and 200 control participants using GenDx NGSgo kit (GenDx, Gaithersburg, MD, USA), Omixon NGS-based HLA holotype kit (Omixon, Budapest, Hungary) and HLA locus-specific primers published previously [18]. Whole-exome capture was performed on samples from 123 patients and 493 control participants using SureSelect XT Human All Exon V5 + UTR or V6 + UTR kit (Agilent Technologies, Santa Clara, CA, USA). High-throughput sequencing was performed on Illumina Miseq and NovaSeq platforms (Illumina, San Diego, CA, USA). A total of 123 patients and 193 control participants were genotyped with both the HLA locus-specific amplification system and the whole-exome capture system, and the HLA genotyping data showed 100% concordance (data not shown). For details on the HLA genotyping, please refer to the ESM Methods.

### Statistical analysis

Comparisons of clinical data between two onset age groups were analysed using SPSS 22.0 software (IBM Corporation, Armonk, NY, USA). Unpaired Student’s t test was used for continuous variables with normal distribution. Mann–Whitney *U* test was used for continuous variables with non-normal distribution. Pearson’s χ^2^ test or Fisher’s exact test was used for categorical variables.

When processing data from whole-exome sequencing, Trim Galore (v.0.3.7) (http://www.bioinformatics.babraham.ac.uk/projects/trim_galore) was used to test quality control on the raw sequencing reads. After the quality control, the sequencing reads were aligned to the hg19 (downloaded from UCSC Genome Browser, http://genome.ucsc.edu/) using BWA-MEM (v.0.7.12) algorithm with default parameters [19]. The extended HLA regional (chr6: 27 Mb–35 Mb) and unmapped reads were extracted by SAMtools (v.1.2) [20]. HLA alleles were assigned by comparing sequencing data with the IPD-IMGT/HLA database (3.28.0) (https://www.ebi.ac.uk/ipd/imgt/hla/) using Omixon HLA Twin (v.2.1.2) (Omixon, Budapest, Hungary). HLA haplotypes were constructed by using BIGDAWG (Bridging ImmunoGenomic Data-Analysis Workflow Gaps) (v.2.1) [21]. All HLA alleles were in Hardy-Weinberg equilibrium in control participants.

Association between HLA genes and type 1 diabetes with different onset ages was analysed using PLINK (v.1.9) (http://pngu.mgh.harvard.edu/~purcell/plink/) [22]. Logistic regression analysis was used to measure the trend-per-copy effect (additive model) of HLA alleles on type 1 diabetes with different onset ages compared with healthy control participants. To investigate the association between HLA genes and onset age of type 1 diabetes, onset age data was double square-root (fourth root) transformed to normal distribution and analysed as a continuous outcome using linear regression. The linear regression of onset age on HLA genes was a case-only analysis, where the HLA genes were encoded by per-haplotype/allele dosage. To test whether the genetic effects of HLA class I alleles were independent of the *DR-DQ* haplotypes, conditional analysis was performed, in which the *DR-DQ* haplotypes were treated as covariates. The *p* value was corrected for multiple comparisons (denoted as *p*c). A *p* or *p*c < 0.05 was considered statistically significant (see the ESM Methods for further details).

## Results

### Overall clinical characteristics of the participants

The characteristics of 361 participants with type 1 diabetes are shown in Table 1. Compared with the <18 group, the ≥18 group had a longer duration of classic symptoms of diabetes before diagnosis (4.3 [IQR, 2.1–12.9] vs 2.9 [IQR 1.4–4.3] weeks), were less likely to have DKA at disease onset (62.72% vs 78.65%) and had lower proportions of positivity for GADA (68.05% vs 77.84%), ZnT8A (11.83% vs 24.86%) and IA-2A (20.71% vs 47.57%). There were no differences in sex ratio, duration of diabetes, fasting and postprandial C-peptide levels and HbA_1c_ levels between the two groups.

**Table 1:** Clinical features of patients with type 1 diabetes in different onset age groups

Characteristic	Total	<18 group	≥18 group	*p* value
*n*	361	192	169	
Sex (M/F)	162/199	78/114	84/85	0.0835
Age at onset (years)	16.6 (10.9–28.0)	11.1 (8.4–14.1)	29.0 (23.3–36.0)	<0.0001
Duration of symptoms pre-diagnosis (weeks)	4.3 (1.4–8.6)	2.9 (1.4–4.3)	4.3 (2.1–12.9)	<0.0001
DKA at onset	257 (71.19)	151 (78.65)	106 (62.72)	0.0009
Duration (years)	1.80 (0.03–5.29)	2.18 (0.01–5.56)	1.49 (0.04–4.96)	0.3922
BMI (kg/m^2^)	19.0 (16.6–21.3)	17.7 (15.4–20.4)	20.2 (18.5–22.1)	<0.0001
GADA+ ^a^	259 (73.16)	144 (77.84)	115 (68.05)	0.0379
ZnT8A+ ^a^	66 (18.64)	46 (24.86)	20 (11.83)	0.0017
IA-2A+ ^a^	123 (34.75)	88 (47.57)	35 (20.71)	<0.0001
Single Ab+ ^a^	169 (47.74)	88 (47.57)	81 (47.93)	0.9458
Multiple Ab+ ^a^	121 (34.18)	82 (44.32)	39 (23.08)	<0.0001
FCP (nmol/l)	0.04 (0.02–0.09)	0.04 (0.02–0.08)	0.04 (0.02–0.09)	0.4118
2 h-PCP (nmol/l) ^b^	0.05 (0.02–0.12)	0.05 (0.02–0.11)	0.05 (0.02–0.15)	0.3951
HbA_1c_ (mmol/mol)	77 (58–107)	79 (60–108)	74 (58–107)	0.5440
HbA_1c_ (%)	9.2 (7.5–11.9)	9.4 (7.6–12.0)	8.9 (7.5–11.9)	0.5373

Data are presented as the median (IQR) for continuous variables, and *n* (%) for categorical variables

^a^Missing data: autoantibody, <18 group, *n* = 7 (1.94%)

^b^Postprandial C-peptide was measured at 2 h during the mixed-meal tolerance test

Abbreviations: 2h-PCP, 2 h postprandial C-peptide; Duration, disease duration at first visit and the duration when islet autoantibodies, C-peptide and HbA_1c_ were measured; F, female; FCP, fasting C-peptide; M, male; Multiple Ab+, positive for two or more islet autoantibodies; Single Ab+, positive for a single islet autoantibody

### Frequency of *DR-DQ* haplotypes in type 1 diabetes with respect to onset age

As shown in Table 2, among patients with onset <18 years, DRB1*03:01:01-DQA1*05:01:01-DQB1*02:01:01 (*DR3*, OR 11.72), DRB1*04:XX-DQA1*03:01:01-DQB1*03:02:01 (XX = 01, 04, 05) (*DR4-DQ8*, OR 11.89), DRB1*04:05:01-DQA1*03:03:01-DQB1*04:01:01 (*DR4-DQ4*, OR 2.35) and DRB1*09:01:02-DQA1*03:02:01-DQB1*03:03:02 (*DR9*, OR 1.65) were predisposing when compared with healthy participants. The frequency of *DR3* (OR 4.99) and *DR4-DQ8* (OR 4.80) was higher in the ≥18 group than in healthy participants. DRB1*11:01:01-DQA1*05:05:01-DQB1*03:01:01 (*DR11*) and DRB1*12:02:01-DQA1*06:01:01-DQB1*03:01:01 (*DR12*) were protective in both the <18 (OR 0.09, 0.09, respectively) and ≥18 (OR 0.40, 0.41, respectively) groups compared with healthy participants.

**Table 2:** Analysis of HLA genes in patients with different onset ages and healthy control participants

HLA genes	<18 group (2*n*= 384) *n* (%)	≥18 group (2*n*= 338) *n* (%)	Control (2*n* = 1000) *n* (%)	<18 group vs Control			≥18 group vs Control			Linear regression between HLA and onset age		
				OR	95% CI	*p*c ^a^	OR	95% CI	*pc* ^a^	β ^b^	SE	*p* value
*DR-DQ* haplotypes												
*DR3*	129 (33.59)	85 (25.15)	61 (6.10)	11.72	7.91, 17.38	<0.0001	4.99	3.42, 7.28	<0.0001	−0.09	0.03	0.0009 *
*DR4-DQ8*	36 (9.38)	15 (4.44)	9 (0.90)	11.89	5.60, 25.25	<0.0001	4.80	2.07, 11.10	0.0005	−0.13	0.05	0.0059 *
*DR4-DQ4*	46 (11.98)	31 (9.17)	57 (5.70)	2.35	1.54, 3.59	0.0001	1.70	1.07, 2.70	0.0520	−0.04	0.04	0.3113
*DR9*	84 (21.88)	54 (15.98)	147 (14.70)	1.65	1.22, 2.24	0.0027	1.11	0.78, 1.56	1.0000	−0.05	0.03	0.0730
*DR11*	2 (0.52)	8 (2.37)	62 (6.20)	0.09	0.02, 0.36	0.0015	0.40	0.19, 0.83	0.0265	0.21	0.09	0.0314
*DR12*	5 (1.30)	19 (5.62)	126 (12.60)	0.09	0.04, 0.23	<0.0001	0.41	0.25, 0.68	0.0011	0.27	0.07	<0.0001 *
*HLA-A* alleles												
A*11:01:01	74 (19.27)	87 (25.74)	323 (32.30)	0.49	0.37, 0.66	<0.0001	0.72	0.54, 0.95	0.0438	0.08	0.03	0.0060
A*24:02:01	85 (22.14)	67 (19.82)	117 (11.70)	2.22	1.61, 3.06	<0.0001	1.94	1.38, 2.74	0.0003	−0.01	0.03	0.6434
A*33:03:01	69 (17.97)	61 (18.05)	84 (8.40)	2.60	1.80, 3.75	<0.0001	2.44	1.69, 3.52	<0.0001	−0.03	0.03	0.3909
*HLA-B* alleles												
B*46:01:01	41 (10.68)	34 (10.06)	176 (17.60)	0.55	0.38, 0.80	0.0031	0.52	0.35, 0.77	0.0024	−0.03	0.04	0.4459
B*54:01:01	41 (10.68)	31 (9.17)	25 (2.50)	5.16	3.04, 8.77	<0.0001	4.27	2.44, 7.47	<0.0001	0.002	0.04	0.9618
B*58:01:01	101 (26.30)	71 (21.01)	73 (7.30)	6.03	4.16, 8.76	<0.0001	3.68	2.52, 5.38	<0.0001	−0.09	0.03	0.0064
*HLA-C* alleles												
C*03:02:02	101 (26.30)	71 (21.01)	67 (6.70)	6.82	4.66, 9.98	<0.0001	4.12	2.80, 6.08	<0.0001	−0.08	0.03	0.0088

^a^Multiplicity adjustments were performed in the comparisons of different onset age groups with control groups (<18 group vs control and ≥ 18 group vs control), where *p*c = *p* × 2 for two pairwise comparisons

^b^The unit for β was double square-root (fourth root) transformed years of change in onset age associated with per copy of the HLA haplotype/allele

*Linear regression result with *p* value <0.05 and with FDR< 5%. The Benjamini–Hochberg FDR correction was conducted according to the number of haplotypes/alleles in each HLA gene (a total of 11 *DR-DQ* haplotypes, 9 *HLA-A* alleles, 9 *HLA-B* alleles and 9 *HLA-C* alleles)

Abbreviations: *DR3*, DRB1*03:01:01-DQA1*05:01:01-DQB1*02:01:01; *DR4-DQ8*, DRB1*04:XX-DQA1*03:01:01-DQB1*03:02:01 (XX = 01, 04, 05); *DR4-DQ4*, DRB1*04:05:01-DQA1*03:03:01-DQB1*04:01:01; *DR9*, DRB1*09:01:02-DQA1*03:02:01-DQB1*03:03:02; *DR11*, DRB1*11:01:01-DQA1*05:05:01-DQB1*03:01:01; *DR12*, DRB1*12:02:01-DQA1*06:01:01-DQB1*03:01:01

Linear regressions of onset age on *DR-DQ* haplotypes show that the *DR3* (β = −0.09) and *DR4-DQ8* (β = −0.13) were negatively associated with onset age, whereas the *DR11* (β = 0.21) and *DR12* (β = 0.27) were positively associated with onset age (Table 2, ESM Table 2 and ESM Fig. 3). The unit for β was double square-root (fourth root) transformed years of change in onset age associated with per copy of the *DR-DQ* haplotype. The distribution of the onset age of patients with different *DR-DQ* haplotypes is shown in ESM Fig. 4. After adjustment for the age-associated clinical metrics shown in Table 1 (duration of symptoms before diagnosis, DKA, BMI and autoantibody prevalence), the associations of *DR3*, *DR4-DQ8* and *DR12* with onset age were still significant (ESM Table 3).

### Effects of HLA class I genes on type 1 diabetes with different onset ages

Frequency data for *HLA-A*, *-B* and *-C* alleles in type 1 diabetes with respect to onset age are shown in Table 2.

The extremely high LD with the strongly associated *DR-DQ* haplotypes could confound the associations of HLA class I genes with the disease. For example, as shown in ESM Fig. 5, A*33:03:01, B*58:01:01, C*03:02:02 and *DR3* were in strong LD with each other. Therefore, in Table 3 and ESM Fig. 6, the associations of HLA class I alleles with type 1 diabetes were adjusted for LD with *DR-DQ* haplotypes. The A*11:01:01 remained positively associated with onset age (β = 0.06), although it showed no significant association with susceptibility to type 1 diabetes. Besides, C*15:02:01 was found negatively associated with onset age (β = −0.21), with an appreciable predisposing effect on patients with onset <18 years when compared with control participants (OR 3.97). The unit for β was double square-root (fourth root) transformed years of change in onset age associated with per copy of the allele. After adjustment for the age-associated clinical metrics shown in Table 1, the associations of A*11:01:01 and C*15:02:01 with onset age were still significant (ESM Table 3).

**Table 3:** Analysis of HLA class I alleles in patients with different onset ages and healthy control participants after adjustment for *DR-DQ* haplotypes

HLA class I alleles	<18 group vs Control			≥18 group vs Control			Linear regression between HLA and onset age		
	OR	95% CI	*p*c ^a^	OR	95% CI	*p*c ^a^	β ^b^	SE	*p* value
*HLA-A* alleles									
A*11:01:01	0.88	0.58, 1.33	1.0000	1.07	0.76, 1.52	1.0000	0.06	0.03	0.0370
A*24:02:01	2.71	1.62, 4.52	0.0003	2.25	1.49, 3.40	0.0002	−0.01	0.03	0.7171
A*33:03:01	0.60	0.30, 1.17	0.2706	1.06	0.62, 1.82	1.0000	0.05	0.04	0.2207
*HLA-B* alleles									
B*46:01:01	0.41	0.23, 0.73	0.0044	0.46	0.28, 0.75	0.0040	−0.01	0.04	0.8498
B*54:01:01	3.96	1.76, 8.91	0.0018	3.79	1.88, 7.66	0.0004	0.06	0.05	0.2075
B*58:01:01	0.65	0.27, 1.60	0.6964	1.10	0.51, 2.36	1.0000	0.02	0.05	0.6375
*HLA-C* alleles									
C*03:02:02	1.19	0.54, 2.62	1.0000	1.78	0.88, 3.62	0.2176	0.03	0.05	0.5875
C*15:02:01	3.97	1.31, 12.01	0.0292	2.26	0.91, 5.60	0.1557	−0.21	0.08	0.0050 *

^a^Multiplicity adjustments were performed in the comparisons of different onset age groups with control groups (<18 group vs control and ≥18 group vs control), where *p*c = *p* × 2 for two pairwise comparisons

^b^The unit for β was double square-root (fourth root) transformed years of change in onset age associated with per copy of the HLA allele

*Linear regression result with *p* value <0.05 and with FDR < 5%. The Benjamini–Hochberg FDR correction was conducted according to the number of alleles in each HLA gene (a total of 9 *HLA-A* alleles, 9 *HLA-B* alleles and 9 *HLA-C* alleles)

A*24:02:01 (OR 2.71, 2.25, respectively) and B*54:01:01 (OR 3.96, 3.79, respectively) were predisposing in both the <18 and ≥18 groups, while B*46:01:01 (OR 0.41, 0.46, respectively) was protective when compared with control participants.

### Effects of HLA class I genes on specific *DR-DQ* haplotypes

Furthermore, we investigated the effects of each HLA class I allele in the context of specific predisposing or protective *DR-DQ* haplotypes. As shown in Table 4, in the <18 group, A*24:02:01 (OR 3.10) and B*54:01:01 (OR 4.36) were associated with increased susceptibility to type 1 diabetes in the context of *DR4-DQ4* when compared with control participants. In addition, the frequency of A*33:03:01-B*58:01:01-C*03:02:02 was lower in the <18 group than in the control group when present on *DR3* (OR 0.43). In the context of *DR11*, C*15:02:01 was increased in the ≥18 group compared with the control group (OR 7.86). In the context of *DR9*, B*54:01:01 enhanced the risk of type 1 diabetes in both the <18 (OR 15.37) and ≥18 (OR 14.90) groups, while B*46:01:01 (OR 0.35, 0.37, respectively) was protective when compared with control participants. In the context of *DR4-DQ4*, A*11:01:01 (61.29% vs 28.26%) significantly increased while A*24:02:01 (19.35% vs 47.83%) significantly decreased in patients with onset ≥18 years when compared with patients with onset <18 years. After adjustment for the age-associated clinical metrics shown in Table 1, in the context of *DR4-DQ4*, A*11:01:01 was still associated with older onset age (*p* = 0.0057), while A*24:02:01 was still associated with younger onset age (*p* = 0.0247).

**Table 4:** Analysis of HLA class I alleles in patients with different onset ages and healthy control participants stratified by *DR-DQ* haplotypes

*DR-DQ*	Class I alleles	<18 group		≥18 group		Control		<18 group vs Control		≥18 group vs Control		<18 group vs ≥18 group
		Class I allele +	% with class I allele	Class I allele +	% with class I allele	Class I allele +	% with class I allele	OR (95% CI)	*p*c ^a^	OR (95% CI)	*p*c ^a^	*p*c ^a^
*DR3*	*A33-B58-C03*	63	48.84	51	60.00	42	68.85	0.43 (0.23, 0.82)	0.0313	0.68 (0.34, 1.36)	0.8209	0.3304
*DR4-DQ4*	A*11:01:01	13	28.26	19	61.29	26	45.61	0.47 (0.21, 1.07)	0.2197	1.89 (0.77, 4.60)	0.4866	0.0144
	A*24:02:01	22	47.83	6	19.35	13	22.81	3.10 (1.33, 7.24)	0.0264	0.81 (0.27, 2.40)	1.0000	0.0403
	B*54:01:01	28	60.87	16	51.61	15	26.32	4.36 (1.89, 10.04)	0.0017	2.99 (1.19, 7.48)	0.0587	1.0000
*DR9*	B*46:01:01	30	35.71	20	37.04	90	61.22	0.35 (0.20, 0.61)	0.0007	0.37 (0.20, 0.71)	0.0080	1.0000
	B*54:01:01	8	9.52	5	9.26	1	0.68	15.37 (1.89, 125.16)	0.0320	14.90 (1.70, 130.65)	0.0443	1.0000
*DR11*	C*15:02:01	0	0.00	4	50.00	7	11.29	–	–	7.86 (1.60, 38.67)	0.0337	–

^a^Multiplicity adjustments were performed in the comparisons of the three groups among each other (<18 group vs control, ≥18 group vs control and <18 group vs ≥18 group), where *p*c = *p* × 3 for three pairwise comparisons

Abbreviations: *DR3*, DRB1*03:01:01-DQA1*05:01:01-DQB1*02:01:01; *DR4-DQ4*, DRB1*04:05:01-DQA1*03:03:01-DQB1*04:01:01; *DR9*, DRB1*09:01:02-DQA1*03:02:01-DQB1*03:03:02; *DR11*, DRB1*11:01:01-DQA1*05:05:01-DQB1*03:01:01; *A33-B58-C03*, A*33:03:01-B*58:01:01-C*03:02:02

### Association of HLA with islet autoantibodies in type 1 diabetes patients

The association between HLA genes and type 1 diabetes-associated autoantibodies was analysed in 139 new-onset (duration of diabetes of less than 1 year) patients (Table 5, ESM Table 4 and ESM Table 5). Compared with GADA negative patients, GADA positive patients had a higher frequency of the high-risk *DR3* (34.94% vs 23.21%) but a lower frequency of the protective *DR12* (1.21% vs 8.04%). A*11:01:01 was negatively associated with GADA (16.27% vs 29.46%), which remained significant after adjustment for *DR3* and *DR12* (data not shown). The frequency of *DR4-DQ8* was higher in ZnT8A positive patients than in ZnT8A negative patients (11.67% vs 2.75%), while *DR3* (40.22% vs 25.27%) and *DR4-DQ8* (9.78% vs 2.15%) were increased in IA-2A positive patients compared with IA-2A negative patients.

**Table 5:** Association of HLA genes with islet autoantibodies in 139 patients with disease duration of less than 1 year

HLA genes	*n* (%)		*p*	Adjusted *p* ^a^
	GADA+ (2*n* = 166)	GADA- (2*n* = 112)		
*DR3*	58 (34.94)	26 (23.21)	0.0306	0.0929
*DR12*	2 (1.21)	9 (8.04)	0.0107	0.0351
A*11:01:01	27 (16.27)	33 (29.46)	0.0113	0.0183
	ZnT8A+ (2*n* = 60)	ZnT8A- (2*n* = 218)		
*DR4-DQ8*	7 (11.67)	6 (2.75)	0.0138	0.0275
	IA-2A+ (2*n* = 92)	IA-2A- (2*n* = 186)		
*DR3*	37 (40.22)	47 (25.27)	0.0089	0.0848
*DR4-DQ8*	9 (9.78)	4 (2.15)	0.0154	0.0393

^a^Adjusted *p*: *p* values for the associations between HLA genes and islet autoantibodies after adjustment for onset age

Abbreviations: *DR3*, DRB1*03:01:01-DQA1*05:01:01-DQB1*02:01:01; *DR4-DQ8*, DRB1*04:XX-DQA1*03:01:01-DQB1*03:02:01 (XX = 01, 04, 05); *DR12*, DRB1*12:02:01-DQA1*06:01:01-DQB1*03:01:01

When the associations were adjusted for onset age, *DR12* and A*11:01:01 were significantly associated with GADA negativity, while *DR4-DQ8* was significantly associated with positivity for ZnT8A and IA-2A.

## Discussion

The genetic risk conferred by HLA genes to type 1 diabetes with different onset ages has not been studied thoroughly. The strong contributions of *HLA-DR* and -*DQ* genes to type 1 diabetes, and the extremely high LD within the HLA region emphasise the importance of adjustment or stratification for *DR-DQ* when analysing the effects of additional susceptibility genes. Our recent study showed that, compared with childhood-onset patients, adult-onset patients had milder clinical features and a lower frequency of high-risk *DR3/DR4* and *DR3/DR9* genotypes [11]. Based on our previous study, we expanded the sample size and did linear regression analyses of *DR-DQ* in patients through all onset ages in the current study. We confirmed that the susceptible *DR3* and *DR4-DQ8* were negatively associated with onset age, while the protective *DR11* and *DR12* were positively associated with onset age. Furthermore, we extended the analysis of HLA genes beyond the classical class II genes to investigate the effects of HLA class I alleles. After adjustment of the data for LD with *DR-DQ* haplotypes, we first reported that A*11:01:01 was positively associated with onset age, while the predisposing C*15:02:01 was negatively associated with onset age. In the context of *DR4-DQ4*, A*11:01:01 was increased, while the predisposing A*24:02:01 was decreased in older onset patients when compared with younger onset patients. These results show that, in addition to *DR-DQ* haplotypes, HLA class I alleles play a role in type 1 diabetes with different onset ages.

Data from the Type 1 Diabetes Genetics Consortium (T1DGC) showed that A*11:01 conferred protection from type 1 diabetes after accounting for LD with *DR-DQ* haplotypes [5]. In this study, the frequency of A*11:01:01 was significantly lower in patients with type 1 diabetes than in healthy participants. However, the protective effect of A*11:01:01 was not significant after adjustment for *DR-DQ* haplotypes. As the frequency of A*11:01:01 was much higher in the Chinese population (32.3%) than in European populations (6.9%) [5], the discrepancy of allele frequency, LD patterns and interactions with environmental factors are possible explanations for different associations of HLA alleles with type 1 diabetes in patients with different ethnicities and genetic backgrounds. In this study, we first reported that A*11:01:01 was positively associated with onset age, which was independent of *DR-DQ* haplotypes. However, further analysis with more stringent correction to control the proportion of type I errors by using false discovery rate (FDR) assessment showed that the FDR for the association of A*11:01:01 with onset age was >5%. Prospective studies and studies with a larger sample size are needed to replicate the results with greater power and explore the causality of the novel associated alleles in affecting onset age.

The frequency of C*15:02:01 was comparable between type 1 diabetes patients and control participants in the unadjusted data. However, after adjustment for LD with *DR-DQ* haplotypes, we first revealed the independent predisposing effect of C*15:02:01, which was stronger in the <18 group than in the ≥18 group. Moreover, the C*15:02 allele was reported to confer risk for ankylosing spondylitis in the East Asian population [23]. We envision that C*15:02 may participate in the pathogenesis of autoimmune-related diseases.

B*54:01:01, which was rarely observed among Europeans [5], has been shown to confer risk for type 1 diabetes in Asian populations [15, 16]. However, those previous studies did not exclude the effect of LD with HLA class II genes on the results, nor did they evaluate it in the context of specific *DR-DQ* haplotypes, so they could not show the independent effect of this allele on type 1 diabetes. In the present study, we showed that, after adjustment for LD with *DR-DQ* haplotypes, B*54:01:01 was significantly associated with increased susceptibility to type 1 diabetes. When stratification of certain HLA class II haplotypes was applied, we first reported that B*54:01:01 significantly enhanced the risk of type 1 diabetes in the context of *DR4-DQ4* and *DR9* haplotypes. These results suggest that HLA class I typing in concert with specific *DR-DQ* genotypes could facilitate genetic prediction of type 1 diabetes. For example, the high OR of B*54:01:01 in the *DR9* haplotype may indicate a significantly increased risk for the individual.

In line with previous studies from Europe and Asia [5, 15, 16], A*24:02:01 was associated with increased susceptibility to type 1 diabetes in our study. Furthermore, A*24:02 was previously reported to be associated with early and complete beta cell destruction [24, 25], and younger onset age of type 1 diabetes [3, 14]. In this study, A*24:02:01 showed no significant association with onset age. However, when analysing the effects of HLA class I alleles on certain *DR-DQ* haplotypes, A*24:02:01 was significantly increased in the <18 group compared with the ≥18 group in the context of the *DR4-DQ4* haplotype. These results suggest that there are interactions between HLA class I and class II alleles and support the previous studies which showed that accumulation of specific HLA class I and class II alleles (including A*24 and DQA1*03) would result in more rapid disease progression [24, 26]. No significant associations between HLA alleles and C-peptide levels were noted in the current study (ESM Table 6 and ESM Table 7), which may be partially due to the low levels of C-peptide in our study. When we classified the patients into three groups according to the stimulated C-peptide concentration levels reported by the DCCT study (‘undetectable’ ≤0.03 nmol/l; ‘minimal’ 0.04–0.20 nmol/l; ‘baseline-only or sustained’ >0.20 nmol/l) [27], we found that the frequency of A*24:02:01 was 20.83% in the ‘undetectable’ group, 22.38% in the ‘minimal’ group and 18.87% in the ‘baseline-only or sustained’ group.

After accounting for the effect of onset age, we found that *DR4-DQ8* was associated with positivity for ZnT8A and IA-2A, which was consistent with earlier findings [28, 29]. In line with this, previous studies suggested that ZnT8A and IA-2A were indicators of more advanced islet autoimmunity and more rapid disease progression that generally appeared closer to the clinical onset of type 1 diabetes [26, 29, 30]. In our study, the frequency of *DR3* was 34.94% in GADA positive patients compared with 23.21% in GADA negative patients, but the difference was not significant when adjusted for onset age (adjusted *p* = 0.0929). Previously, *DR3* has been shown to be associated with GADA. A recent study found that GAD65-specific immunotherapy had a significant effect on C-peptide retention in individuals with recent-onset type 1 diabetes who had the *DR3-DQ2* haplotype, suggesting that antigen-specific immunotherapy may be most effective when targeting a specific HLA haplotype/allele linked to the tolerising antigen [31]. With increasing focus on disease heterogeneity in evaluating therapeutic strategies, our results may support using HLA genotyping to identify appropriate immunotherapies. HLA-A*24 has been shown to be negatively associated with IA-2A independently of the primary class II effects [32]. In our data, the effect of A*24:02:01 on IA-2A was not significant after conditioning on the leading HLA class II associations and onset age (OR 0.58, *p* = 0.1632), whereas A*11:01:01 was found negatively associated with GADA. Based on these findings, further studies would be needed to examine the association between autoantibodies and HLA class I alleles. Whether class I alleles could influence the effects of immunomodulatory therapies for type 1 diabetes may now get more attention.

It should be noted that the patients with onset ≥18 years included in our study were clearly distinguished from those with LADA, who are characterised by the presence of autoantibodies, adult age of diagnosis and preserved beta cell function at the time of diagnosis. Previous studies have indicated that, compared with type 1 diabetes, the effect sizes of type 1 diabetes risk HLA haplotypes in LADA were smaller, while the impact of type 1 diabetes protective HLA haplotypes was greater in LADA [33, 34]. Moreover, these differences were apparent even when comparing LADA patients to adult-onset type 1 diabetes patients [33]. A recent study showed that in LADA, the independent effects of HLA class I observed in type 1 diabetes were not observed after conditioning on the leading HLA class II associations, suggesting that the HLA class I association may be a genetic discriminator between LADA and type 1 diabetes [35].

There are some limitations to our study. We observed a high percentage of DKA and female/male ratio in our study. Interestingly, previous studies suggested that female sex was associated with an increased risk of DKA [36–39]. We suspect that this may be due to patient selection. Although analyses with adjustment for DKA or sex indicate that DKA and sex ratio have no significant effect on our analysis of type 1 diabetes–HLA association (ESM Table 8 and ESM Table 9), the potential impact of DKA and sex ratio in the overall population still need to be confirmed with more studies. In addition, because the discrepancy of allele frequency, LD patterns and interactions with environmental factors may influence the associations of HLA alleles with type 1 diabetes in patients with different genetic backgrounds, the age-associated HLA class I alleles detected in this study may not be generalised to other ethnicities. Besides, given the strong impact of diabetes duration on antibody prevalence and C-peptide levels, we restricted the analyses of associations between HLA genes and autoantibodies to patients with a disease duration of less than 1 year and adjusted the analysis on C-peptide for diabetes duration. However, we cannot completely rule out the potential influence of disease duration.

In conclusion, in addition to the well-studied effects of the HLA class II genes, we further investigated the effects of HLA class I genes on type 1 diabetes with different onset ages in a Chinese population, which could improve the understanding of disease heterogeneity and has implications for the design of recruitment strategies for future studies.

## Supplementary Information

ESM (PDF 566 kb)

## Data Availability

All data of the current study are available from the corresponding author upon reasonable request.

## Abbreviations

DKA: Diabetic ketoacidosis
FDR: False discovery rate
GADA: GAD autoantibodies
IA-2A: Insulinoma-associated antigen-2 autoantibody
LADA: Latent autoimmune diabetes in adults
LD: Linkage disequilibrium
ZnT8A: Zinc transporter 8 autoantibodies
