# Supplementary material for: HLA class I genes modulate disease risk and age at onset together with DR-DQ in Chinese patients with insulin-requiring type 1 diabetes
Source: Diabetologia. 2021 May 22;64(9):2026–36. doi: 10.1007/s00125-021-05476-6 (PMC8382651; doi:10.1007/s00125-021-05476-6)
Supplement: Supplementary file 1 — (PDF 566 kb) [file 125_2021_5476_MOESM1_ESM.pdf]

Electronic supplementary material:

HLA class I genes modulate disease risk and age at onset together with *DR-DQ* in Chinese patients with insulin-requiring type 1 diabetes

Methods

Genotyping methods The *HLA-A*, *-B*, *-C*, *-DR*, and *-DQ* loci are distinguished by their extraordinary polymorphism and the extensive linkage disequilibrium. To ensure the accuracy and reliability of our data, we used three methods for next-generation sequencing (NGS)-based HLA typing in this study, i.e.

1) Commercial NGS-based HLA genotyping kit

   Amplicons of HLA genes were generated in samples from 24 patients in the <18 group using GenDx NGSgo kit (GenDx, Gaithersburg, MD, USA. *HLA-A*, *-B*, *-C* and *-DQA1* primers spanning 5'UTR to 3'UTR, *HLA-DRB1* primers spanning exon 2 to exon 3, *HLA-DQB1* primers spanning exon 2 to exon 4).

   Amplicons of HLA genes were generated in samples from 123 patients in the <18 group, 51 patients in the ≥18 group and 200 healthy control participants using Omixon NGS-based HLA holotype kit (Omixon, Budapest, Hungary. *HLA-A*, *-B*, *-C* and *-DQA1* primers spanning 5'UTR to 3'UTR, *HLA-DRB1* primers spanning exon 2 to exon 4, *HLA-DQB1* primers spanning exon 2 to exon 6).

2) The published HLA locus-specific amplification method [1]

   Amplicons of HLA genes were generated in samples from 192 patients in the <18 group, 169 patients in the ≥18 group and 193 healthy control participants using primers published previously (*HLA-A*, *-B*, *-C*, *-DQA1* and *-DQB1* primers spanning enhancer-promotor to 3'UTR, *HLA-DRB1* set 1 primers spanning enhancer-promoter to exon 2 and set 2 primers spanning exon2 to 3'UTR).

3) Whole-exome sequencing system

   Whole-exome capture was performed on samples from 72 patients in the <18 group using SureSelect XT Human All Exon V5+UTR kit (Agilent Technologies, Santa Clara, CA, USA).

   Whole-exome capture was performed on samples from 51 patients in the <18 group and 493 healthy control participants using SureSelect XT Human All Exon V6+UTR kit (Agilent Technologies, Santa Clara, CA, USA).

After getting the libraries by the above three methods, high-throughput sequencing was performed on Illumina Miseq and NovaSeq platform (Illumina, San Diego, CA, USA).

123 patients in the <18 group and 193 healthy control participants were genotyped with all the above three methods, and the genotyping data showed 100% concordance.

Statistical analysis For the analyses where multiplicity adjustments were required to perform to control the experimentwise error rate, pc = raw p value × n (n separate tests are performed) and raw p value was not shown in the table. A $pc < 0.05$ was considered statistically significant.

For the analyses where adjustments for multiple tests were unnecessary, $p < 0.05$ was considered statistically significant.

ESM Table 1 Power calculation for 361 patients with type 1diabetes and 500 healthy control participants

OR / Freq	**1.3**	**1.4**	**1.5**	**1.6**	**1.7**	**1.8**	**1.9**	**2.0**	**2.1**	**2.2**	**2.3**	**3.0**
**0.05**	0.2400	0.3726	0.5170	0.6543	0.7699	0.8573	0.9173	0.9551	0.9770	0.9889	>0.9900	>0.9900
**0.10**	0.4010	0.6049	0.7766	0.8916	0.9545	0.9833	>0.9900	>0.9900	>0.9900	>0.9900	>0.9900	>0.9900
**0.15**	0.5231	0.7460	0.8920	0.9629	0.9896	>0.9900	>0.9900	>0.9900	>0.9900	>0.9900	>0.9900	>0.9900
**0.20**	0.6112	0.8281	0.9425	0.9852	>0.9900	>0.9900	>0.9900	>0.9900	>0.9900	>0.9900	>0.9900	>0.9900
**0.25**	0.6730	0.8759	0.9657	>0.9900	>0.9900	>0.9900	>0.9900	>0.9900	>0.9900	>0.9900	>0.9900	>0.9900
**0.30**	0.7152	0.9037	0.9768	>0.9900	>0.9900	>0.9900	>0.9900	>0.9900	>0.9900	>0.9900	>0.9900	>0.9900
**0.35**	0.7426	0.9195	0.9823	>0.9900	>0.9900	>0.9900	>0.9900	>0.9900	>0.9900	>0.9900	>0.9900	>0.9900
**0.40**	0.7585	0.9277	0.9848	>0.9900	>0.9900	>0.9900	>0.9900	>0.9900	>0.9900	>0.9900	>0.9900	>0.9900

Power calculation was performed by using Genetic Power Calculator (https://zzz.bwh.harvard.edu/gpc/). Number of type 1 diabetes cases, 361; number of healthy control participants, 500; significance level, 0.05. Conditions with statistical power ≥ 80% are marked in gray

ESM Table 2 Power calculation for significant haplotypes/alleles in linear regression of onset age in Table 2

HLA genes	Power
DR3	0.9174
DR4-DQ8	0.7917
DR11	0.5799
DR12	0.9822
*A*11:01:01*	0.7897
*B*58:01:01*	0.7841
*C*03:02:02*	0.7497

Power calculation was performed by using PASS v.15 (NCSS, Kaysville, UT, USA). Sample size, 361; significance level, 0.05

ESM Table 3 Linear regression between HLA genes and onset age with adjustment for duration of symptoms before diagnosis, DKA, BMI and autoantibody prevalence

HLA genes	β [a]	SE	*p* value	Adjust for *DR-DQ*		
				β [a]	SE	*p* value
DR3	-0.07	0.03	0.0107	-	-	-
DR4-DQ8	-0.08	0.04	0.0454	-	-	-
DR11	0.07	0.08	0.3947	-	-	-
DR12	0.13	0.06	0.0465	-	-	-
*A*11:01:01*	0.06	0.03	0.0196	0.06	0.03	0.0469
*C*15:02:01*	-0.16	0.06	0.0138	-0.21	0.07	0.0031

[a] The unit for β was double square-root (fourth root) transformed years of change in onset age associated with per copy of the HLA haplotype/allele

Abbreviations: *DR3*, *DRB1*03:01:01-DQA1*05:01:01-DQB1*02:01:01*; *DR4-DQ8*, *DRB1*04:XX-DQA1*03:01:01-DQB1*03:02:01 (XX = 01, 04, 05)*; *DR11*, *DRB1*11:01:01-DQA1*05:05:01-DQB1*03:01:01*; *DR12*, *DRB1*12:02:01-DQA1*06:01:01-DQB1*03:01:01*

ESM Table 4 Clinical features of patients with disease duration of less than 1 year in different onset age groups

Characteristic	Total	<18 group	≥18 group	*p* value
n	139	69	70	
Sex (M/F)	70/69	31/38	39/31	0.2035
Age at onset (years)	18.0 (11.0–29.5)	11.0 (8.5–13.8)	29.4 (23.2–37.2)	<0.0001
Duration of symptoms pre-diagnosis (weeks)	4.3 (1.2–5.5)	2.9 (1.0–4.3)	4.3 (2.0–8.6)	0.0322
DKA at onset	91 (65.47)	54 (78.26)	37 (52.86)	0.0016
Duration (years)	0 (0–0.18)	0 (0–0.06)	0.02 (0–0.21)	0.2578
BMI(kg/m^2)	18.1 (16.0–20.7)	16.0 (14.1–18.1)	20.0 (18.0–22.1)	<0.0001
GADA+	83 (59.71)	50 (72.46)	33 (47.14)	0.0023
ZnT8A+	30 (21.58)	23 (33.33)	7 (10.00)	0.0008
IA-2A+	46 (33.09)	36 (52.17)	10 (14.29)	<0.0001
Single Ab+	47 (33.81)	24 (34.78)	23 (32.86)	0.8104
Multiple Ab+	47 (33.81)	35 (50.72)	12 (17.14)	<0.0001
FCP (nmol/l)	0.07 (0.03–0.12)	0.08 (0.04–0.14)	0.07 (0.03–0.10)	0.1974
2h-PCP (nmol/l) [a]	0.11 (0.04–0.23)	0.12 (0.07–0.25)	0.10 (0.04–0.23)	0.2583
HbA_{1c} (mmol/mol)	83 (56–124)	91 (61–122)	80 (55–132)	0.8895
HbA_{1c} (%)	9.7 (7.3–13.5)	10.5 (7.7–13.3)	9.5 (7.2–14.2)	0.8825

Data are presented as the median (IQR) for continuous variables, and *n* (%) for categorical variables

[a] Postprandial C-peptide was measured at 2 h during the mixed-meal tolerance test

Abbreviations: 2h-PCP, 2 h postprandial C-peptide; Duration, disease duration at first visit and the duration when islet autoantibodies, C-peptide and HbA_{1c} were measured; F, female; FCP, fasting C-peptide; M, male; Multiple Ab+, positive for two or more islet autoantibodies; Single Ab+, positive for a single islet autoantibody

ESM Table 5 Association of HLA genes with islet autoantibody status in 139 patients with disease duration of less than 1 year

HLA genes	*n* (%)		*p*	Adjusted *p* [a]
	Ab+ (2*n* = 188)	Ab- (2*n* = 90)		
DR3	65 (34.57)	19 (21.11)	0.0182	0.1062
DR12	2 (1.06)	9 (10.00)	0.0024	0.0194
	Multiple Ab+ (2*n* = 94)	Multiple Ab- (2*n* = 184)		
DR3	38 (40.43)	46 (25.00)	0.0068	0.0592
DR4-DQ8	10 (10.64)	3 (1.63)	0.0057	0.0159

[a] Adjusted *p*: *p* values for the associations between HLA genes and islet autoantibodies after adjustment for onset age

Abbreviations: Ab+, positive for at least one islet autoantibody; Ab-, positive for no islet autoantibody; *DR3*, *DRB1*03:01:01-DQA1*05:01:01-DQB1*02:01:01*; *DR4-DQ8*, *DRB1*04:XX-DQA1*03:01:01-DQB1*03:02:01 (XX = 01, 04, 05)*; *DR12*, *DRB1*12:02:01-DQA1*06:01:01-DQB1*03:01:01*; Multiple Ab+, positive for two or more islet autoantibodies; Multiple Ab-, positive for one or no islet autoantibody

ESM Table 6 Linear regression between HLA genes and C-peptide levels with adjustment for onset age, duration of diabetes at C-peptide testing and BMI

HLA genes	*n* (%)	β	SE	*p* value
DR-DQ haplotypes				
DR3	209 (29.69)	-0.02	0.02	0.4300
DR4-DQ8	49 (6.96)	0.04	0.03	0.2189
DR4-DQ4	77 (10.94)	-0.05	0.03	0.0871
DR7	30 (4.26)	-0.01	0.04	0.7395
DR8	11 (1.56)	-0.03	0.06	0.6259
DR9	132 (18.75)	-0.02	0.02	0.3808
DR11	10 (1.42)	-0.02	0.07	0.7527
DR12	23 (3.27)	-0.01	0.05	0.8262
DR15	13 (1.85)	-0.03	0.06	0.6250
DR16	16 (2.27)	0.10	0.06	0.1131
HLA-A alleles				
*A*02:01:01*	49 (6.96)	0.02	0.04	0.4992
*A*02:03:01*	31 (4.40)	0.03	0.04	0.4160
*A*02:06:01*	19 (2.70)	-0.01	0.06	0.8146
*A*02:07:01*	78 (11.08)	-0.02	0.03	0.4684
*A*11:01:01*	159 (22.59)	0.001	0.02	0.9612
*A*11:02:01*	15 (2.13)	0.05	0.06	0.3893
*A*24:02:01*	149 (21.16)	-0.01	0.02	0.5413
*A*26:01:01*	19 (2.70)	-0.01	0.05	0.9110
*A*33:03:01*	126 (17.90)	-0.01	0.02	0.5764
HLA-B alleles				
*B*13:01:01*	23 (3.27)	-0.04	0.05	0.4538
*B*15:02:01*	51 (7.24)	-0.03	0.04	0.3741
*B*38:02:01*	15 (2.13)	-0.05	0.06	0.4702

*B*40:01:02*	100 (14.20)	-0.004	0.02	0.8770
*B*46:01:01*	74 (10.51)	0.01	0.03	0.6124
*B*51:01:01*	24 (3.41)	0.08	0.05	0.0795
*B*54:01:01*	70 (9.94)	-0.05	0.03	0.1381
*B*55:02:01*	13 (1.85)	0.10	0.07	0.1285
*B*58:01:01*	167 (23.72)	-0.01	0.02	0.8014
HLA-C alleles				
*C*01:02:01*	130 (18.47)	-0.02	0.02	0.3761
*C*03:02:02*	167 (23.72)	-0.01	0.02	0.7538
*C*03:03:01*	20 (2.84)	-0.01	0.05	0.8857
*C*03:04:01*	63 (8.95)	-0.02	0.03	0.4714
*C*07:02:01*	107 (15.20)	-0.02	0.02	0.4842
*C*08:01:01*	74 (10.51)	-0.03	0.03	0.3748
*C*12:02:02*	12 (1.71)	0.06	0.07	0.3861
*C*14:02:01*	19 (2.70)	0.004	0.06	0.9373
*C*15:02:01*	19 (2.70)	0.07	0.05	0.1643

Abbreviations: *DR3*, *DRB1*03:01:01-DQA1*05:01:01-DQB1*02:01:01*; *DR4-DQ8*, *DRB1*04:XX-DQA1*03:01:01-DQB1*03:02:01 (XX = 01, 04, 05)*; *DR4-DQ4*, *DRB1*04:05:01-DQA1*03:03:01-DQB1*04:01:01*; *DR7*, *DRB1*07:01:01-DQA1*02:01:01-DQB1*02:02:01*; *DR8*, *DRB1*08:03:02-DQA1*01:03:01-DQB1*06:01:01*; *DR9*, *DRB1*09:01:02-DQA1*03:02:01-DQB1*03:03:02*; *DR11*, *DRB1*11:01:01-DQA1*05:05:01-DQB1*03:01:01*; *DR12*, *DRB1*12:02:01-DQA1*06:01:01-DQB1*03:01:01*; *DR15*, *DRB1*15:01:01-DQA1*01:02:01-DQB1*06:01:01*; *DR16*, *DRB1*16:02:01-DQA1*01:02:02-DQB1*05:02:01*

ESM Table 7 Logistic regression between HLA genes and C-peptide levels with adjustment for onset age, duration of diabetes at C-peptide testing and BMI

HLA genes	Baseline	Minimal	Undetectable	Baseline vs Minimal			Baseline vs Undetectable			Minimal vs Undetectable		
	n (%)	*n* (%)	*n* (%)	OR	95% CI	*pc* [a]	OR	95% CI	*pc* [a]	OR	95% CI	*pc* [a]
DR-DQ haplotypes												
DR3	30 (28.30)	80 (27.97)	99 (31.73)	0.88	0.46, 1.68	1.0000	0.72	0.32, 1.62	1.0000	0.78	0.51, 1.18	0.7086
DR4-DQ8	7 (6.60)	19 (6.64)	23 (7.37)	1.54	0.56, 4.21	1.0000	2.48	0.61, 10.15	0.6150	1.69	0.80, 3.59	0.5091
DR4-DQ4	7 (6.60)	36 (12.59)	34 (10.90)	0.47	0.18, 1.24	0.3759	0.43	0.14, 1.34	0.4413	1.04	0.58, 1.89	1.0000
DR7	5 (4.72)	14 (4.90)	11 (3.53)	0.76	0.21, 2.73	1.0000	0.46	0.08, 2.57	1.0000	1.05	0.45, 2.46	1.0000
DR8	1 (0.94)	5 (1.75)	5 (1.60)	0.58	0.07, 4.66	1.0000	0.29	0.02, 3.36	0.9573	0.96	0.28, 3.35	1.0000
DR9	18 (16.98)	55 (19.23)	59 (18.91)	0.83	0.42, 1.64	1.0000	1.00	0.46, 2.18	1.0000	0.97	0.62, 1.50	1.0000
DR11	0 (0.00)	6 (2.10)	4 (1.28)	-	-	-	-	-	-	2.22	0.53, 9.37	0.8286
DR12	3 (2.83)	10 (3.50)	10 (3.21)	1.46	0.30, 6.95	1.0000	1.69	0.25, 11.21	1.0000	1.36	0.45, 4.07	1.0000
DR15	1 (0.94)	5 (1.75)	7 (2.24)	0.73	0.06, 8.68	1.0000	0.32	0.03, 3.62	1.0000	0.93	0.26, 3.39	1.0000
DR16	6 (5.66)	4 (1.40)	6 (1.92)	1.39	0.30, 6.42	1.0000	0.74	0.13, 4.04	1.0000	0.47	0.12, 1.82	0.8259
HLA-A alleles												
*A*02:01:01*	8 (7.55)	19 (6.64)	22 (7.05)	1.25	0.43, 3.65	1.0000	1.56	0.38, 6.42	1.0000	1.13	0.55, 2.33	1.0000
*A*02:03:01*	5 (4.72)	10 (3.50)	16 (5.13)	1.36	0.34, 5.40	1.0000	0.56	0.14, 2.25	1.0000	0.62	0.26, 1.48	0.8397
*A*02:06:01*	7 (6.60)	5 (1.75)	7 (2.24)	2.87	0.69, 11.85	0.4365	1.29	0.32, 5.28	1.0000	0.43	0.12, 1.61	0.6309
*A*02:07:01*	14 (13.21)	31 (10.84)	33 (10.58)	1.53	0.68, 3.47	0.9210	1.53	0.57, 4.10	1.0000	1.03	0.58, 1.83	1.0000
*A*11:01:01*	19 (17.92)	67 (23.43)	73 (23.40)	0.87	0.47, 1.63	1.0000	0.87	0.42, 1.77	1.0000	1.05	0.69, 1.61	1.0000
*A*11:02:01*	4 (3.77)	2 (0.70)	9 (2.89)	3.90	0.62, 24.58	0.4416	0.90	0.18, 4.50	1.0000	0.21	0.04, 1.05	0.1717
*A*24:02:01*	20 (18.87)	64 (22.38)	65 (20.83)	0.64	0.33, 1.24	0.5550	1.26	0.56, 2.80	1.0000	1.14	0.72, 1.80	1.0000
*A*26:01:01*	4 (3.77)	11 (3.85)	4 (1.28)	1.01	0.26, 3.99	1.0000	1.41	0.26, 7.80	1.0000	2.13	0.69, 6.55	0.5604
*A*33:03:01*	17 (16.04)	52 (18.18)	57 (18.27)	0.91	0.44, 1.89	1.0000	0.75	0.33, 1.68	1.0000	0.90	0.55, 1.48	1.0000
HLA-B alleles												
*B*13:01:01*	2 (1.89)	7 (2.45)	14 (4.49)	0.82	0.14, 4.92	1.0000	0.26	0.04, 1.74	0.4941	0.57	0.21, 1.54	0.8031
*B*15:02:01*	7 (6.60)	21 (7.34)	23 (7.37)	1.46	0.48, 4.39	1.0000	0.58	0.17, 1.95	1.0000	1.07	0.51, 2.21	1.0000

*B*38:02:01*	4 (3.77)	3 (1.05)	8 (2.56)	1.75	0.30, 10.37	1.0000	0.50	0.09, 2.71	1.0000	0.31	0.07, 1.30	0.3294
*B*40:01:02*	11 (10.38)	44 (15.38)	45 (14.42)	0.60	0.28, 1.29	0.5685	0.67	0.27, 1.61	1.0000	0.96	0.58, 1.59	1.0000
*B*46:01:01*	15 (14.15)	25 (8.74)	34 (10.90)	1.42	0.66, 3.05	1.0000	2.53	0.97, 6.65	0.1772	0.89	0.47, 1.68	1.0000
*B*51:01:01*	5 (4.72)	9 (3.15)	10 (3.21)	1.69	0.42, 6.74	1.0000	1.89	0.31, 11.43	1.0000	1.22	0.43, 3.50	1.0000
*B*54:01:01*	4 (3.77)	31 (10.84)	35 (11.22)	0.29	0.08, 0.98	0.1393	0.34	0.09, 1.28	0.3348	0.87	0.47, 1.59	1.0000
*B*55:02:01*	4 (3.77)	4 (1.40)	5 (1.60)	3.18	0.64, 15.74	0.4704	4.75	0.53, 42.72	0.4929	0.68	0.14, 3.25	1.0000
*B*58:01:01*	25 (23.58)	67 (23.43)	75 (24.04)	1.00	0.50, 1.98	1.0000	0.95	0.40, 2.24	1.0000	0.91	0.57, 1.44	1.0000
HLA-C alleles												
*C*01:02:01*	18 (16.98)	55 (19.23)	57 (18.27)	0.78	0.39, 1.56	1.0000	1.20	0.52, 2.77	1.0000	1.07	0.65, 1.74	1.0000
*C*03:02:02*	25 (23.58)	65 (22.73)	77 (24.68)	1.08	0.54, 2.17	1.0000	0.83	0.37, 1.87	1.0000	0.81	0.51, 1.29	1.0000
*C*03:03:01*	4 (3.77)	8 (2.80)	8 (2.56)	1.23	0.31, 4.94	1.0000	3.68	0.49, 27.35	0.6102	1.12	0.41, 3.08	1.0000
*C*03:04:01*	8 (7.55)	20 (6.99)	35 (11.22)	1.18	0.44, 3.18	1.0000	0.47	0.16, 1.36	0.4971	0.59	0.32, 1.11	0.3066
*C*07:02:01*	13 (12.26)	42 (14.69)	52 (16.67)	0.60	0.28, 1.31	0.6027	0.70	0.29, 1.64	1.0000	0.74	0.46, 1.20	0.6693
*C*08:01:01*	7 (6.60)	34 (11.89)	33 (10.58)	0.85	0.32, 2.23	1.0000	0.54	0.17, 1.73	0.9105	1.42	0.78, 2.59	0.7620
*C*12:02:02*	4 (3.77)	7 (2.45)	1 (0.32)	1.69	0.40, 7.10	1.0000	18.26	0.82, 405.70	0.1991	8.04	0.88, 73.24	0.1929
*C*14:02:01*	4 (3.77)	8 (2.80)	7 (2.24)	1.09	0.26, 4.59	1.0000	1.74	0.26, 11.87	1.0000	1.76	0.51, 6.01	1.0000
*C*15:02:01*	3 (2.83)	8 (2.80)	8 (2.56)	0.95	0.15, 6.08	1.0000	1.31	0.08, 21.46	1.0000	1.33	0.47, 3.74	1.0000

[a] Multiplicity adjustments were performed in the comparisons of the three groups among each other (baseline vs minimal, baseline vs undetectable and minimal vs undetectable), where $pc = p\times 3$ for three pairwise comparisons

Abbreviations: *DR3*, *DRB1*03:01:01-DQA1*05:01:01-DQB1*02:01:01*; *DR4-DQ8*, *DRB1*04:XX-DQA1*03:01:01-DQB1*03:02:01 (XX = 01, 04, 05)*; *DR4-DQ4*, *DRB1*04:05:01-DQA1*03:03:01-DQB1*04:01:01*; *DR7*, *DRB1*07:01:01-DQA1*02:01:01-DQB1*02:02:01*; *DR8*, *DRB1*08:03:02-DQA1*01:03:01-DQB1*06:01:01*; *DR9*, *DRB1*09:01:02-DQA1*03:02:01-DQB1*03:03:02*; *DR11*, *DRB1*11:01:01-DQA1*05:05:01-DQB1*03:01:01*; *DR12*, *DRB1*12:02:01-DQA1*06:01:01-DQB1*03:01:01*; *DR15*, *DRB1*15:01:01-DQA1*01:02:01-DQB1*06:01:01*; *DR16*, *DRB1*16:02:01-DQA1*01:02:02-DQB1*05:02:01*

ESM Table 8 Linear regression between HLA genes and onset age with adjustment for DKA at disease onset

HLA genes	β [a]	SE	*p* value	Adjust for *DR-DQ*		
				β [a]	SE	*p* value
DR3	-0.10	0.03	0.0004	-	-	-
DR4-DQ8	-0.13	0.05	0.0042	-	-	-
DR11	0.21	0.09	0.0225	-	-	-
DR12	0.27	0.07	<0.0001	-	-	-
*A*11:01:01*	0.08	0.03	0.0037	0.06	0.03	0.0306
*C*15:02:01*	-0.09	0.07	0.2206	-0.20	0.08	0.0074

[a] The unit for β was double square-root (fourth root) transformed years of change in onset age associated with per copy of the HLA haplotype/allele

Abbreviations: *DR3*, *DRB1*03:01:01-DQA1*05:01:01-DQB1*02:01:01*; *DR4-DQ8*, *DRB1*04:XX-DQA1*03:01:01-DQB1*03:02:01 (XX = 01, 04, 05)*; *DR11*, *DRB1*11:01:01-DQA1*05:05:01-DQB1*03:01:01*; *DR12*, *DRB1*12:02:01-DQA1*06:01:01-DQB1*03:01:01*

ESM Table 9 Linear regression between HLA genes and onset age with adjustment for sex

HLA genes	β [a]	SE	*p* value	Adjust for *DR-DQ*		
				β [a]	SE	*p* value
DR3	-0.09	0.03	0.0011	-	-	-
DR4-DQ8	-0.12	0.05	0.0094	-	-	-
DR11	0.21	0.09	0.0265	-	-	-
DR12	0.27	0.07	<0.0001	-	-	-
*A*11:01:01*	0.08	0.03	0.0059	0.06	0.03	0.0370
*C*15:02:01*	-0.10	0.07	0.1523	-0.21	0.08	0.0049

[a] The unit for β was double square-root (fourth root) transformed years of change in onset age associated with per copy of the HLA haplotype/allele

Abbreviations: *DR3*, *DRB1*03:01:01-DQA1*05:01:01-DQB1*02:01:01*; *DR4-DQ8*, *DRB1*04:XX-DQA1*03:01:01-DQB1*03:02:01 (XX = 01, 04, 05)*; *DR11*, *DRB1*11:01:01-DQA1*05:05:01-DQB1*03:01:01*; *DR12*, *DRB1*12:02:01-DQA1*06:01:01-DQB1*03:01:01*

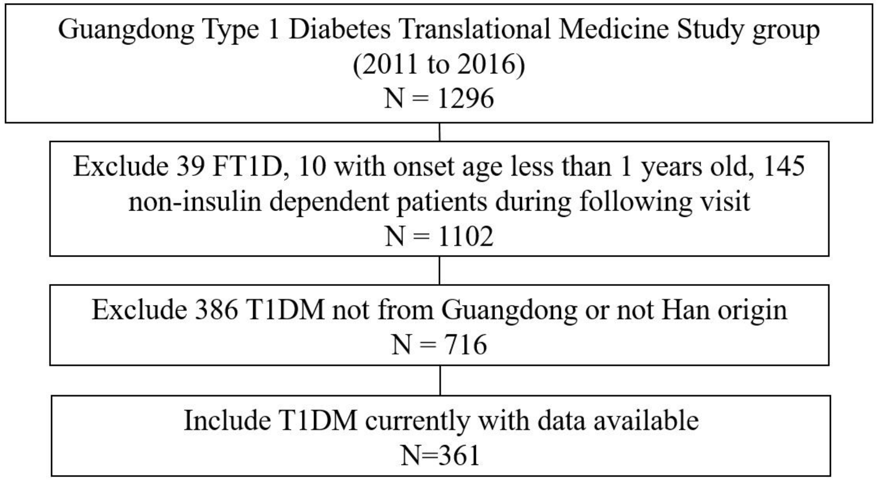

ESM Fig. 1 Patient selection of this study. A total of 1296 patients with type 1 diabetes were enrolled in the Guangdong Type 1 Diabetes Translational Medicine Study between 2011 and 2016. During the follow-up, 155 patients were considered not to have type 1 diabetes and were excluded (10 were less than 1 year old at the onset of diabetes, and 145 were not insulin-dependent during the following visits). Additional exclusions included 39 patients with fulminant type 1 diabetes and 386 patients who were non-Guangdong residents or non-Han Chinese. We ascertained 716 cases with a final diagnosis of type 1 diabetes and Han Chinese origin. In addition to the 214 cases of classical type 1 diabetes which have been reported previously [2], we expanded the sample size without setting additional selection criteria to restrict DKA or autoantibody status. As a result, a total of 361 patients currently with data available were included in this study

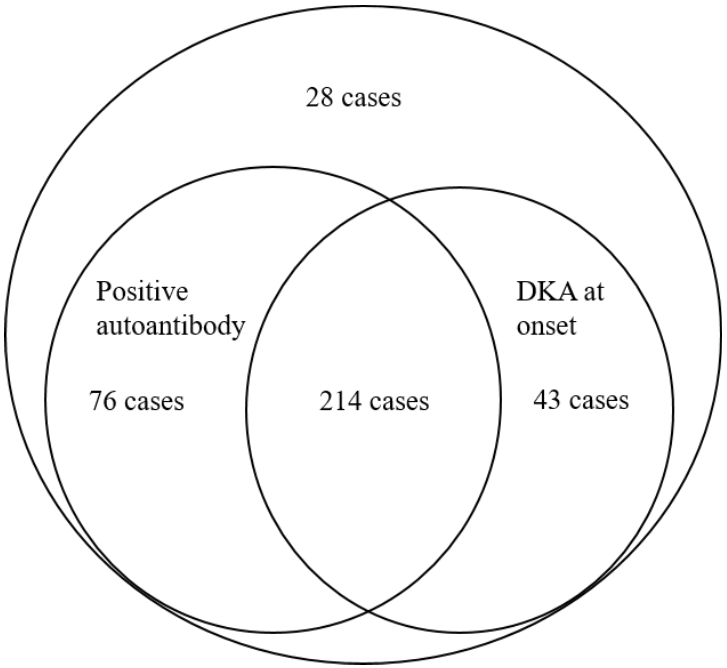

ESM Fig. 2 Venn diagram of 361 patients with type 1 diabetes included in this study showing subsets of patients with positive autoantibody and DKA. 290 patients were tested positive for at least one islet autoantibody. 257 patients presented with DKA at disease onset

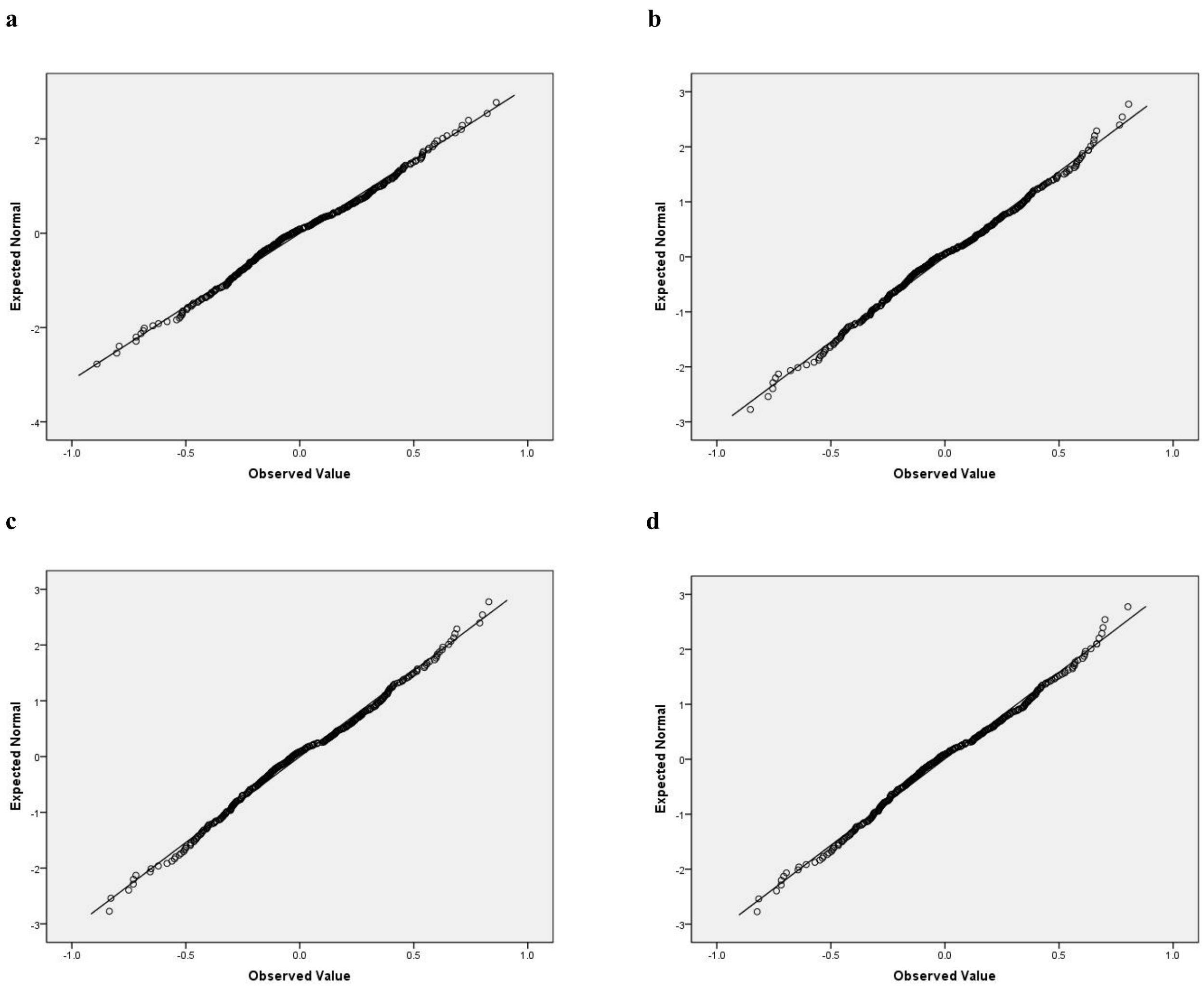

ESM Fig. 3 Normal Q-Q plots of regression residuals in linear regressions of onset age on *DR3* (**a**), *DR4-DQ8* (**b**), *DR11* (**c**) and *DR12* (**d**). Shapiro-Wilk tests of normality shown that regression residuals were approximately normal

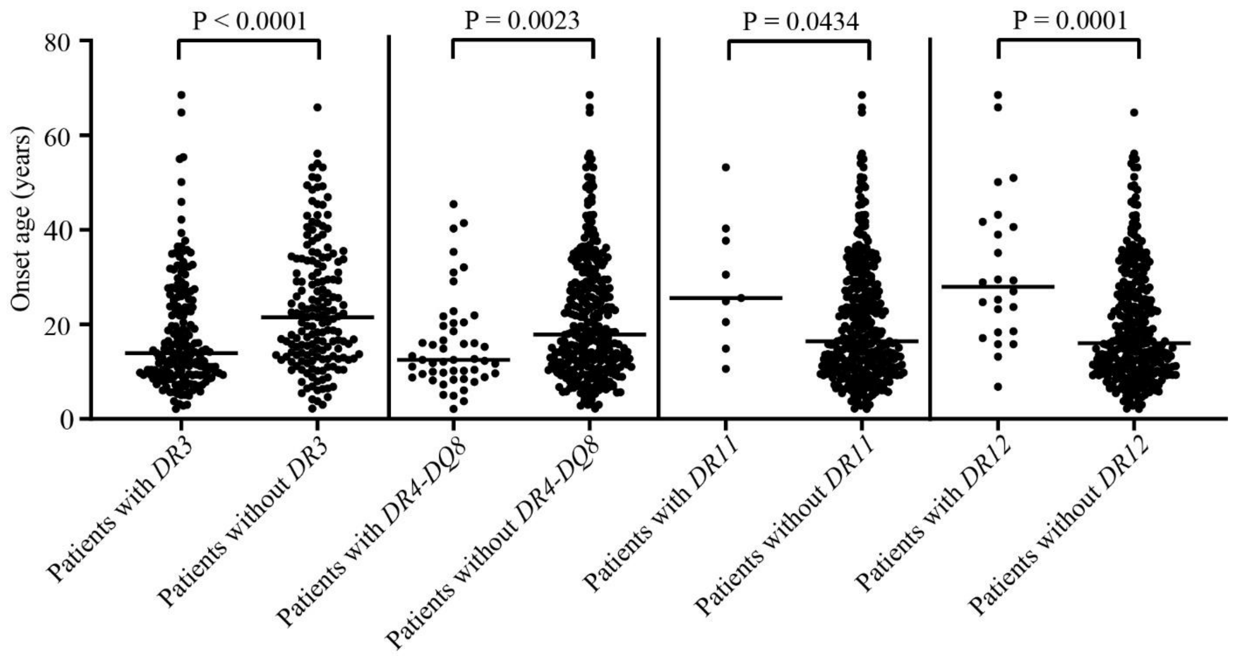

ESM Fig. 4 Distribution of onset age of patients with different *DR-DQ* haplotypes. Scatter plots of onset age with a bar indicting the median

a

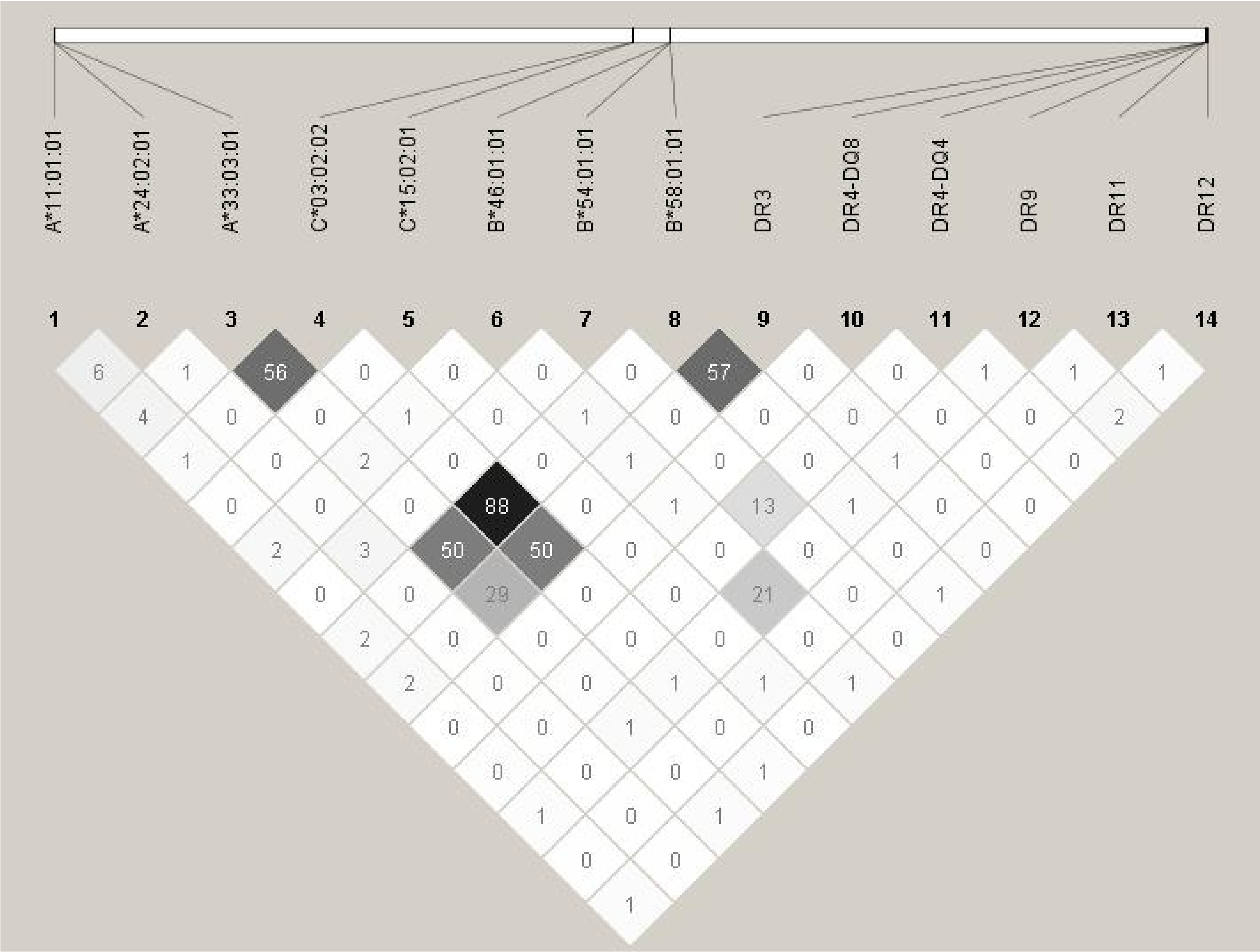
A*11:01:01
A*24:02:01
A*33:03:01
C*03:02:02
C*15:02:01
B*46:01:01
B*54:01:01
B*58:01:01
DR3
DR4-DQ8
DR4-DQ4
DR9
DR11
DR12
1
2
3
4
5
6
7
8
9
10
11
12
13
14

b

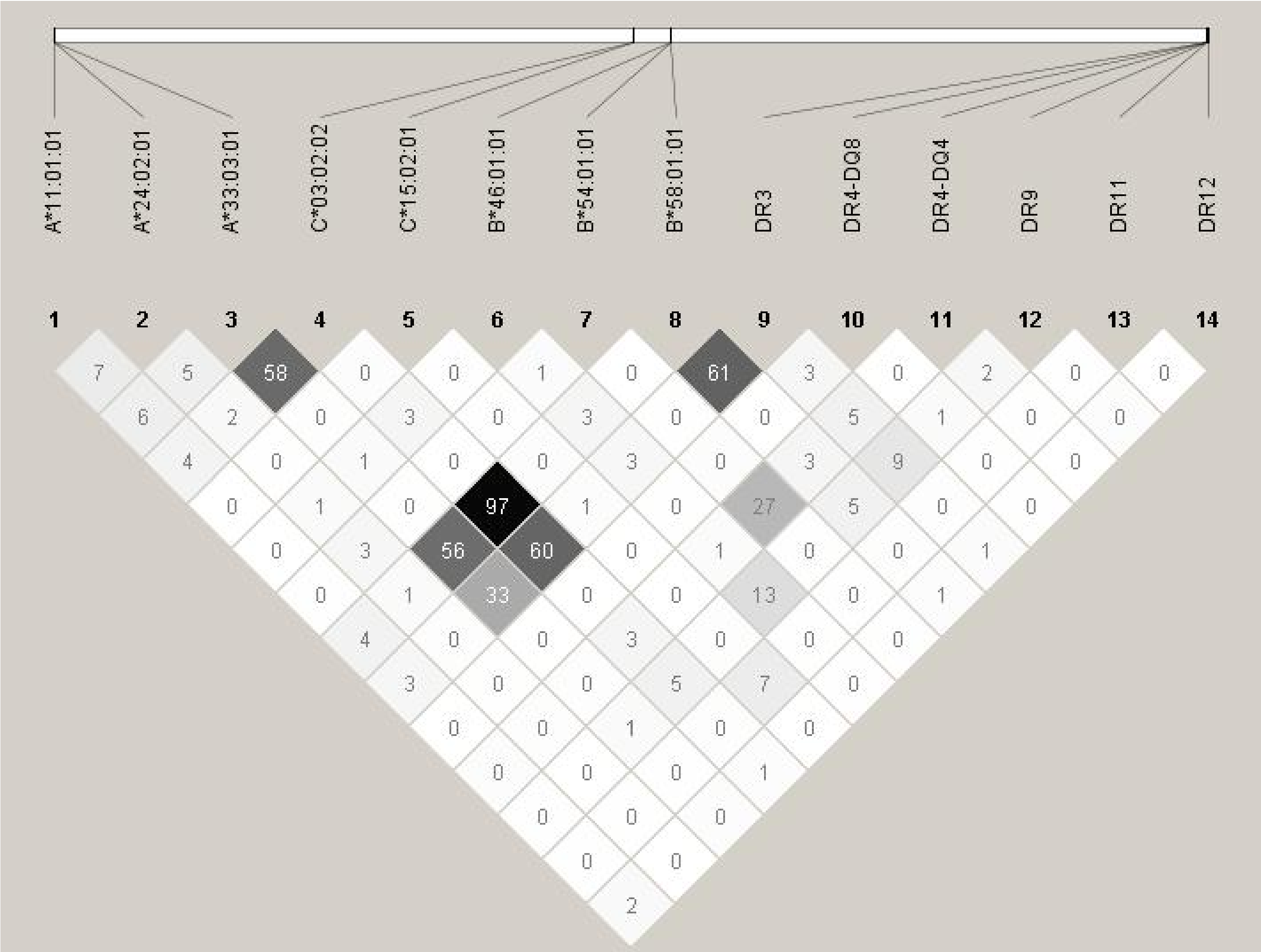
A*11:01:01
A*24:02:01
A*33:03:01
C*03:02:02
C*15:02:01
B*46:01:01
B*54:01:01
B*58:01:01
DR3
DR4-DQ8
DR4-DQ4
DR9
DR11
DR12

ESM Fig. 5 LD diagrams for *HLA-A*, *-C*, *-B* alleles and *DR-DQ* haplotypes in 500 healthy control participants (**a**) and 361 patients with type 1 diabetes (**b**). The $r^2 \times 100$ values of LD between pairs of alleles were shown. The main haplotype block included *A*33:03:01*, *B*58:01:01*, *C*03:02:02* and *DR3*

Abbreviations: *DR3*, *DRB1*03:01:01-DQA1*05:01:01-DQB1*02:01:01*; *DR4-DQ8*, *DRB1*04:XX-DQA1*03:01:01-DQB1*03:02:01 (XX = 01, 04, 05)*; *DR4-DQ4*, *DRB1*04:05:01-DQA1*03:03:01-DQB1*04:01:01*; *DR9*, *DRB1*09:01:02-DQA1*03:02:01-DQB1*03:03:02*; *DR11*, *DRB1*11:01:01-DQA1*05:05:01-DQB1*03:01:01*; *DR12*, *DRB1*12:02:01-DQA1*06:01:01-DQB1*03:01:01*

a b

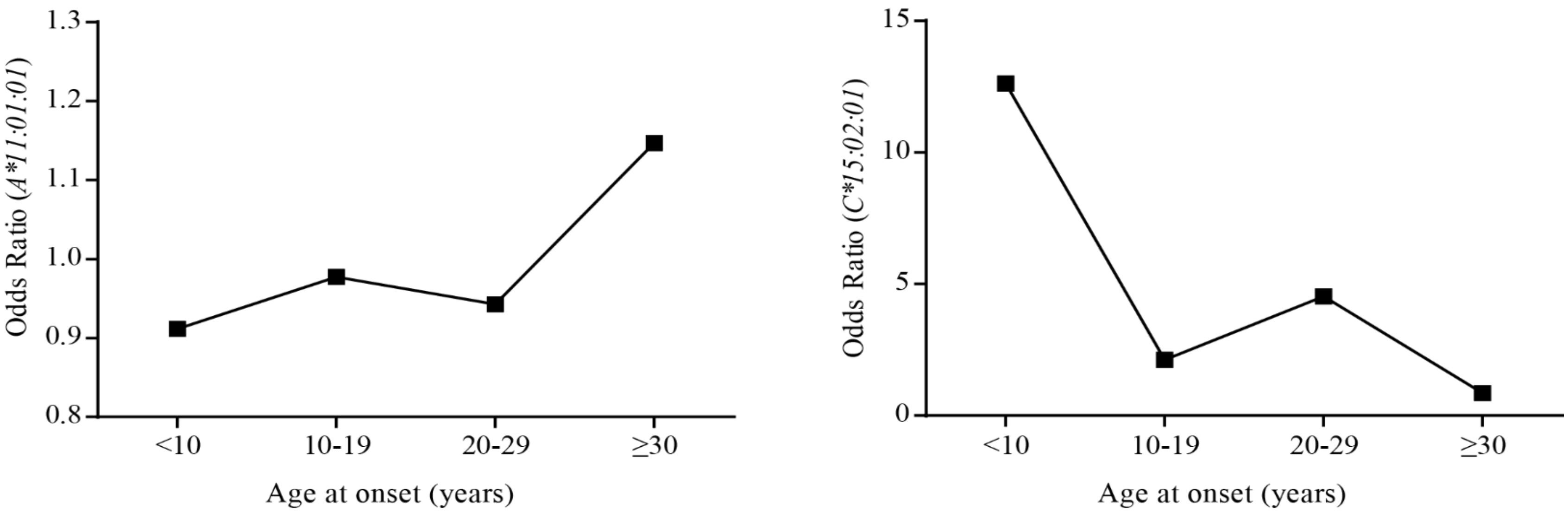

ESM Fig. 6 The odds ratios for *A*11:01:01* (**a**) and *C*15:02:01* (**b**) after adjustment for LD with *DR-DQ* in type 1 diabetes patients with different onset ages

Reference

1. Shiina T, Suzuki S, Ozaki Y et al (2012) Super high resolution for single molecule-sequence-based typing of classical HLA loci at the 8-digit level using next generation sequencers. Tissue Antigens 80(4): 305-316. https://doi.org/10.1111/j.1399-0039.2012.01941.x
2. Ren W, Yang D, Jiang Z et al (2020) Adult-onset type 1 diabetic patients with less severe clinical manifestation have less risk DR-DQ genotypes than childhood-onset patients. Diabetes Metab Res Rev: e3357. https://doi.org/10.1002/dmrr.3357
